# C_60_ Fullerene as an Effective Nanoplatform of Alkaloid Berberine Delivery into Leukemic Cells

**DOI:** 10.3390/pharmaceutics11110586

**Published:** 2019-11-08

**Authors:** Anna Grebinyk, Svitlana Prylutska, Anatoliy Buchelnikov, Nina Tverdokhleb, Sergii Grebinyk, Maxim Evstigneev, Olga Matyshevska, Vsevolod Cherepanov, Yuriy Prylutskyy, Valeriy Yashchuk, Anton Naumovets, Uwe Ritter, Thomas Dandekar, Marcus Frohme

**Affiliations:** 1 Division Molecular Biotechnology and Functional Genomics, Technical University of Applied Sciences Wildau, Hochschulring 1, 15745 Wildau, Germany; grebinyk@th-wildau.de (A.G.); sgrebinyk@th-wildau.de (S.G.); 2 Department of Bioinformatics, Biocenter, University of Würzburg, Am Hubland, 97074 Würzburg, Germany; dandekar@biozentrum.uni-wuerzburg.de; 3 Taras Shevchenko National University of Kyiv, Volodymyrska 64, 01601 Kyiv, Ukraine; psvit_1977@ukr.net (S.P.); prylut@ukr.net (Y.P.); yashchukvaleriy@gmail.com (V.Y.); 4 Laboratory of Molecular and Cell Biophysics, Sevastopol State University, 299053 Sevastopol, Crimea; buchelnikov@bsu.edu.ru (A.B.); tverdokhlebnm@gmail.com (N.T.); max_evstigneev@mail.ru (M.E.); 5 Laboratory of Organic Synthesis and NMR Spectroscopy, Belgorod State University, 308015 Belgorod, Russia; 6 Palladin Institute of Biochemistry, NAS of Ukraine, Leontovicha Str. 9, 01030 Kyiv, Ukraine; matysh@yahoo.com; 7 Institute of Physics, NAS of Ukraine, 46 av. Nauki, 03028 Kyiv, Ukraine; vvch2000@ukr.net (V.C.); agn@iop.kiev.ua (A.N.); 8 Institute of Chemistry and Biotechnology, University of Technology Ilmenau, Weimarer Straße 25 (Curiebau), 98693 Ilmenau, Germany; uwe.ritter@tu-ilmenau.de

**Keywords:** C_60_ fullerene, Berberine, noncovalent nanocomplex, UV–Vis, DLS and AFM measurements, drug release, leukemic cells, uptake, cytotoxicity, apoptosis

## Abstract

A herbal alkaloid Berberine (Ber), used for centuries in Ayurvedic, Chinese, Middle-Eastern, and native American folk medicines, is nowadays proved to function as a safe anticancer agent. Yet, its poor water solubility, stability, and bioavailability hinder clinical application. In this study, we have explored a nanosized carbon nanoparticle—C_60_ fullerene (C_60_)—for optimized Ber delivery into leukemic cells. Water dispersions of noncovalent C_60_-Ber nanocomplexes in the 1:2, 1:1, and 2:1 molar ratios were prepared. UV–Vis spectroscopy, dynamic light scattering (DLS), and atomic force microscopy (AFM) evidenced a complexation of the Ber cation with the negatively charged C_60_ molecule. The computer simulation showed that π-stacking dominates in Ber and C_60_ binding in an aqueous solution. Complexation with C_60_ was found to promote Ber intracellular uptake. By increasing C_60_ concentration, the C_60_-Ber nanocomplexes exhibited higher antiproliferative potential towards CCRF-CEM cells, in accordance with the following order: free Ber < 1:2 < 1:1 < 2:1 (the most toxic). The activation of caspase 3/7 and accumulation in the sub-G1 phase of CCRF-CEM cells treated with C_60_-Ber nanocomplexes evidenced apoptosis induction. Thus, this study indicates that the fast and easy noncovalent complexation of alkaloid Ber with C_60_ improved its in vitro efficiency against cancer cells.

## 1. Introduction

Historically natural products have always provided drugs against a wide variety of diseases, with cancer being no exception [1]. Herbal secondary metabolites exhibit multiple biological and pharmacological properties, representing a natural library of bioactive compounds with potentially high safety, availability, accessibility, and low costs. Alkaloids, being one of the most versatile class of herbal secondary metabolites, are heterocyclic, nitrogen-containing, low-molecular-weight molecules that provide plants with a defense against herbivores, bacteria, fungi, and viruses [2,3,4]. Representatives of this class often exhibit pharmacological effects and are used as anticancer therapeutics such as Vinblastine, Vincristine, Paclitaxel, and Camptothecin [5].

The isoquinoline quaternary alkaloid Berberine (Ber: 2,3-methylenedioxy-9,10-dimethoxyprotoberberine chloride, CAS No. 2086-83-1) is a common drug in Ayurvedic, Chinese, Middle-Eastern, and native American folk medicines [6,7] due to its broad spectra of biological activities. Ber applications as a low-cost therapeutic with anti-inflammatory, antimutagenic [8], antidiabetic [9], antimicrobial, and antiviral effects seem to be promising [4,10]. In recent years, Ber has been reported to inhibit the proliferation of many cancer cell lines originated from head and neck squamous carcinoma [11], melanoma [12], leukemia [3,13,14], oral [15], pancreatic [16], colon [17], breast [18], and prostate cancer [19]. Given the nitrogen atom positive charge [20], Ber interacts directly with genomic [21,22] and telomeric [23,24] DNA, inducing double-strand breaks, and telomere stabilization, respectively. The other potential intracellular targets of Ber are DNA topoisomerase I, POT1 [3], Wnt [17,25], p53 [18,26], NF-kB [27], cyclooxygenase-2, Mcl-1 [15], nucleophosmin/B23 [13], and death-domain-associated protein [28]. Ber’s anticancer cytotoxicity is associated mainly with oxidative stress escalation and mitochondrial dysfunction [7,16,19], apoptosis activation, and cell cycle arrest [10,14,15,18,28]. 

The antiproliferative properties raise a possibility for its use as an anticancer therapeutic agent; however, the poor water solubility, stability, and bioavailability [29] limit its clinical applications. Furthermore, Ber’s hormetic effect [30], when low doses strongly stimulate the growth of cancer cells, and high doses have an anticancer effect, challenges the suitable dosage range. Delivery nanosystems could provide a means of overcoming limitations and improving Ber’s anticancer efficacy. The advent of nanomedicine and application of biocompatible, bioavailable, and nontoxic nanoparticles has brought significant advances in the field of cancer therapy, offering a customizable and safer treatment option.

C_60_ fullerene (C_60_, CAS No. 99685-96-8) [31], a third allotropic form of carbon, has a stable spherical-like hollow structure with a 0.72 nm diameter, the surface of which consists of 60 carbon atoms. C_60_ is a highly efficient “free radical sponge” [32] due to pronounced electrophilicity (the ability to accept up to six electrons). Wang et al. [33] reported that C_60_ and its derivatives efficiently prevent peroxidation and membrane breakdown triggered by free radical species and are more effective in inhibiting lipid peroxidation than a natural antioxidant vitamin E. C_60_ is a hydrophobic molecule able to penetrate into both the lipid bilayer and the cell membranes [34,35]. Pristine C_60_ [36,37] and its water-soluble derivative [38] were found to be accumulated in mitochondria.

The pristine C_60_ has a very low solubility in water. However, it can form a stable aqueous colloid solution (C_60_FAS), which contains both individual C_60_ molecules and its nanoaggregates [39,40]. C_60_ is active only in a soluble form when its carbon double bounds are freely accessible [41]. Recently we have demonstrated that C_60_FAS prevented the restraint stress-induced oxidative disorders in rats’ brain and heart tissues [42] as well as CCl_4_-induced acute liver injury [43], more effectively diminished the muscle fatigue in rats comparable to the known exogenous antioxidants N-acetylcysteine or β-alanine [44], markedly decreased the oxidative stress and enhanced the activity of antioxidant enzymes in rats with diet-induced obesity [45], had anti-inflammatory and hepatoprotective effects in a model of acute colonic inflammation [46], and protected the heart and liver of tumor-bearing mice against Doxorubicin-induced oxidative stress [47].

It is important to note that pristine C_60_ is not toxic against normal cells at low concentrations: according to our previous data [48,49], C_60_FAS at concentrations up to 14.4 as well as 24 µg/mL did not manifest any toxic effects in rat erythrocytes and thymocytes as well as in human mesenchymal stem cells, respectively. The estimation of C_60_FAS impact on *Drosophila melanogaster* at DNA, tissue, and organism levels showed that C_60_ at the concentration 40 μg/mL does not affect the reproductive system and embryogenesis [50]. Recently, a low toxicity of C_60_FAS towards human embryonic kidney (HEK293) cells and mice (IC_50_ 383.4 μg/mL, LD_50_ 721 mg/kg) [51] and the selective strong toxic effect of C_60_FAS against tumor cells (rat and human glioma cells) and transformed human phagocytes [52] were demonstrated.

It is known that anticancer drugs used in clinical practice (including gold standards Doxorubicin and Cisplatin) have long been characterized by a high adverse toxicity. Reducing the side effects of these drugs can be achieved by creating an effective targeted delivery nanosystem based, for example, on biocompatible and bioavailable C_60_ [53]. It was previously shown that, when Doxorubicin or Cisplatin were immobilized on C_60_ fullerene, their intracellular concentration in cancer cells was increased, leading to a pronounced antitumor effect in in vitro and in vivo systems [34,54,55,56,57,58,59,60]. Thus, one can suggest that C_60_ complexation with a traditional drug is a promising nanoformulation for targeted drug delivery, substantially increasing its medico-biological effectiveness with a novel dosage form in the subsequent preclinical screening [61,62].

**Figure 1:**
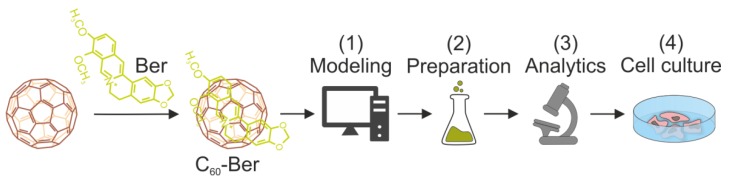
The workflow for the development of C_60_-based cancer chemotherapy with the use of Berberine (Ber): (**1**) computer modeling of the C_60_-Ber nanocomplex; (**2**) the fast and cost-effective preparation of nanocomplexes in different molar ratios in aqueous solutions; (**3**) the analytical assessment of nanocomplex stability to prove its biological applicability; (**4**) noncovalent complexation of aromatic Ber molecule with C_60_, improving its efficiency against human leukemic cells.

The purpose of this study (Figure 1) was to test the formation of the C_60_-Ber nanocomplex in an aqueous solution using computer simulation and physico-chemical characterization. The nanocomplex was designed in three molar ratios of C_60_ to Ber—1:2, 1:1, and 2:1—to investigate whether C_60_’s concentration affected complexation efficacy and Ber bioactivity. Finally, the nanocomplex was applied towards cancer cells in vitro to study whether complexation with C_60_ affects Ber’s intracellular accumulation and cytotoxic potential.

Leukemic cell lines are common models of human cancer for experimental investigations at the cellular level. Leukemia, cancer of the body’s blood-forming tissues, including bone marrow and the lymphatic system, reached 437,033 in terms of new diagnosed cases in 2018, which is an average of 14 out of 100,000 persons per year. Thus, the human leukemic CCRF-CEM cells were chosen as a main in vitro cancer model for the current research.

## 2. Materials and Methods

### 2.1. Chemicals

Roswell Park Memorial Institute medium (RPMI 1640), phosphate buffered saline (PBS), fetal bovine serum (FBS), penicillin/streptomycin, l-glutamin, and Trypsin were obtained from Biochrom (Berlin, Germany). 3-(4,5-dimethylthiazol-2-yl)-2,5-Diphenyl tetrazolium bromide (MTT), ethanol, triton X-100, RNAse A, propidium iodide, and Ber were obtained from Sigma-Aldrich Co. (St-Louis, MO, USA). Dimethylsulfoxide (DMSO) and trypan blue from Carl Roth GmbH + Co. KG (Karlsruhe, Germany) were used.

### 2.2. Preparation of C_60_ with a Ber Aqueous Solution

The highly stable purified C_60_FAS (>99.5%, concentration 2.6 mg/mL) was prepared by ultrasonication of toluene dissolved C_60_ in aqueous phase [40].

Cationic Ber [C_20_H_18_NO_4_]^+^ was dissolved in distilled water with an initial concentration of 1 mg/mL.

C_60_FAS and Ber were mixed in various molar ratios, namely, C_60_-Ber as 1:1 (208:208 µM), 1:2 (208:416 µM), and 2:1 (208:104 µM). The resulting C_60_+Ber dispersions were treated in the ultrasonic disperser for 20 min and afterwards stirred magnetically for 18 h at room temperature.

The working concentrations of the C_60_-Ber nanocomplexes used for cell treatment are presented in the following Ber equivalent concentrations in order to compare the effect of nanocomplexes with the effect of the free alkaloid at the same concentration.

### 2.3. UV–Vis Spectroscopy

UV–Vis absorption spectra of freshly prepared C_60_-Ber dispersions were recorded using a double-beam spectrophotometer SQ-4802 (Unico, Waltham, MA, USA) at room temperature. The measurements were performed using quartz cells with an optical path length of 1 cm in the range of 200–500 nm. Initially, we put a mixture of C_60_FAS (the concentration in the mixture was 0.016 mM) and Ber (the concentration in the mixture was 0.02 mM) into a cell. In order to maintain the Ber concentration, constant titration was accomplished using a Ber solution of the same concentration by sequential dilution of the initial mixture down to zero C_60_ concentration. Since C_60_FAS has a pronounced spectrum in the UV-region, overlapping with the Ber spectrum and containing scattering in the whole spectral region, it was necessary to exclude it by applying differential measurements [57]; i.e., C_60_FAS at a concentration of 0.016 mM was placed in a reference cell and diluted simultaneously with the dilution of the mixture.

### 2.4. AFM Measurement

The atomic force microscopy (AFM) was performed to determine the intermolecular interactions and the degree of components’ aggregation in layers of the free components (Ber and C_60_) and their nanocomplex (C_60_-Ber). AFM measurements were done with the “Solver Pro M” system (NT-MDT, Moscow, Russia). A drop of investigated solution was transferred on the atomically smooth substrate to deposit layers. Measurements were carried out after complete evaporation of the solvent. A freshly broken surface of mica (SPI supplies, V-1 grade) was used as a substrate. Measurements were carried out in a semicontact (tapping) mode with AFM probes of the RTPESPA150 (Bruker, 6 N/m, 150 kHz) type.

### 2.5. DLS Measurement

The intensity size distribution and the values of the polydispersity index (PDI) and the zeta potential for various freshly prepared aqueous systems containing different particles were determined by dynamic light scattering (DLS) on a Zetasizer Nano-ZS90 (Malvern, Worcestershire, UK) at room temperature. The instrument was equipped with a He-Ne laser (5 mW) operating at a wavelength of 633 nm. The autocorrelation function of the scattered light intensity was analyzed by the Malvern Zetasizer software with Smoluchowski approximation. The size distribution was used to calculate the mean hydrodynamic diameter.

### 2.6. Ber Release with HPLC-ESI-MS/MS

C_60_-Ber nanocomplexes were incubated in RPMI over 72 h under the identical conditions adopted from cell-based experiments (2 mL, 37 °C). For sample purification from a released free drug, 500 µL of each sample was filtered with the centrifugal filter devices Amicon Ultra-0.5 3 K (Sigma-Aldrich Co., St-Louis, MO, USA) according to the manufacturer’s instructions: 14,000× *g*, 15 min for filtration; 1000× *g*, 2 min for recovery (reverse spin upside down in a new centrifuge tube).

**Figure 2:**
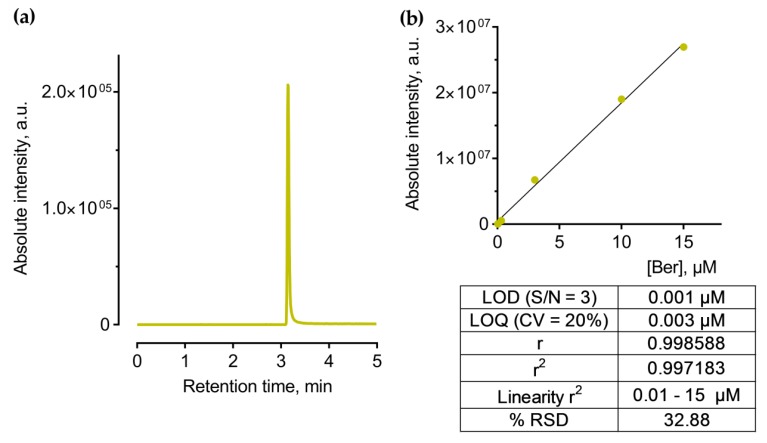
Data of the developed HPLC-ESI-MS method for Ber detection and quantification: representative MRM-chromatogram of Ber (**a**) and a calibration curve with the method’s performance characteristics used for drug content quantification: LOD—limit of detection, S/N—signal/noise ratio, LOQ—limit of quantitation, RSD—relative standard deviation (**b**).

The content of the filter device was subjected to the chromate-mass spectrometry. Elution and separation of Ber was performed using the Eclipse XDB-C18 column under gradient conditions with a mobile phase of methanol and a 0.1% formic acid water solution. The following linear gradient elution was used: 5% B held for 0.5 min, then increased to 100% from 0.5 to 3.5 min, then held at 100% B from 3.5 to 4 min, then decreased to 5% B from 4 to 4.5 min, and further held at 5% B for 5 min. The flow rate was set at 0.7 mL/min. The chromatographic reverse phase conditions and optimized MS/MS parameters are presented in Table 1. For identification and quantification, the molecular ion of Ber was chosen (Figure 2a).

HPLC-ESI-MS/MS analysis was performed in positive mode with usage of multiple reactions monitoring (MRM) mode, which provides the best sensitivity and accuracy of measurements. After MS/MS optimization, a unique MRM-transition that includes a precursor and two characteristic product ions was acquired and used for further identification and quantification. The ionized Ber molecule ([M]^+^, 336.25 *m/z*) was used as a precursor ion with the most abundant fragment ions of 321.20, 320.20, and 292.25 *m/z*.

Ber calibration standards from 0.03 to 15 μg/mL were prepared from a 30 μg/mL water stock solution. These standards were stored in the dark at 40 °C. Quantification was achieved using the regression curve (Figure 2b) according to the linear regression Equation (1):

*y* = (5,37328e + 006)*x* + 389633. (1)

The obtained data were normalized with the RPMI control and expressed as a percentage of the respective control sample, analyzed at 0 h.

### 2.7. Computer Simulation

The computation of spatial structures as well as the computation of component binding energies related to particular physical factors were accomplished using an approach similar to that employed previously in the analysis of C_60_ complexation with various small molecules [57,61].

Structures of the C_60_ and Ber molecule were taken from the Protein Data Bank 32 [63] (PDB codes Ids C_60_ and Ber, respectively). The structure of the C_60_-Ber nanocomplex was calculated by the methods of molecular mechanics using X-PLOR, version 3.1 [64]. The atomic charges were calculated using Gaussian 03W [65]. The topology and parametrization of their valent interactions were obtained with the help of XPLO2D software [66].

The intramolecular van der Waals energy of the C_60_-Ber complexation was calculated using X-PLOR. The calculation of electrostatic energy was performed by solution of the nonlinear Poisson-Boltzmann equation (the NLPB method) using DelPhi [67]. The computation of hydrophobic energy was performed based on a linear correlation between the hydrophobic dissolution energy and a variation of the solvent accessible surface areas (ΔSASA) as Δ*G*_hyd_ = *γ*·ΔSASA, where *γ* = 50 cal/(mol·Å^2^) is a microscopic surface tension coefficient. SASA was calculated using GETAREA, version 1.1 [68].

### 2.8. Cell Culture

The human cancer T-cell line CCRF-CEM (ACC 240) of leucosis origin was purchased from the Leibniz Institute DSMZ-German Collection of Microorganisms and Cell Cultures (Deutsche Sammlung von Mikroorganismen und Zellkulturen, Braunschweig, Germany). CCRF-CEM cells were maintained in an RPMI 1640 medium, supplemented with 10% FBS, 1% penicillin/streptomycin, and 2 mM glutamine. Cells were cultured in 25 cm^2^ culture flasks at a 37 °C with 5% CO_2_ in a humidified incubator binder (Tuttlingen, Germany). The passaging was performed once cells reached ≈80%. Treatment with Trypsin (1:10 in PBS) was used to detach adherent cells. The number of viable cells was counted upon 0.1% trypan blue staining with a Roche Cedex XS analyzer (Basel, Switzerland).

### 2.9. Flow Cytometry

Two 10^5^ CCRF-CEM cells, incubated in 6-well plates for 24 h, were treated with 10 µM free and C_60_ bound Ber. After 0, 1, 3, and 6 h incubation, cells per sample were analyzed at λ_ex_ = 488 nm and λ_em_ = 530/40 nm with the flow cytometer BD FACSJazz™ (Franklin Lakes, NJ, USA).

### 2.10. Fluorescent Microscopy

CCRF-CEM cells were incubated with 10 µM Ber and C_60_-Ber nanocomplexes in a Ber equivalent concentration. At 0, 1, 3, and 6 h, the cells were examined with a Keyence BZ-9000 BIOREVO fluorescence microscope (Osaka, Japan), equipped with a green filter (λ_ex_ = 435 nm, λ_em_ > 515 nm). The Keyence BZ-II Viewer acquisition software (Osaka, Japan) was used.

### 2.11. Cell Viability Assay

CCRF-CEM cells, cultured in 96-well cell culture plates Sarstedt (Nümbrecht, Germany) for 24 h, were treated with the 1% FBS medium containing 0–80 µM Ber or C_60_-Ber nanocomplexes in a Ber equivalent concentration. Cell viability was determined with an MTT reduction assay [69] at 24, 48, and 72 h. Briefly, cells were incubated for 2 h at 37 °C in the presence of 0.5 mg/mL MTT. The diformazan crystals were dissolved in DMSO and determined at 570 nm with a microplate reader Tecan Infinite M200 Pro (Männedorf, Switzerland).

### 2.12. Cell Cycle

CCRF-CEM cells (2 × 10^5^/well, 2 mL) were seeded in 6-well plates, incubated for 24 h, and subsequently treated with 10 µM free and C_60_-bound Ber. After 12 h incubation, the cells were harvested, washed with PBS, fixed by adding the cell solution dropwise to ice-cold 70% ethanol/PBS, mixed, and stored at 20 °C overnight. Next, cells were washed with ice-cold PBS and treated with the working buffer containing 100 µg/mL RNAse A (in water, preboiled at 95 °C for 15 min), 0.1% triton X-100, and 10 µg/mL propidium iodide for 20 min. Consequently, the DNA content of cells was analyzed with the BD FACSJazz™ flow cytometer (Franklin Lakes, NJ, USA). A minimum of 2 × 10^4^ events per sample were acquired and analyzed at λ_ex_ = 488 nm and λ_em_ = 692/40 nm with BD FACS™ (Franklin Lakes, NJ, USA).

### 2.13. Caspase 3/7 Activity

CCRF-CEM cells (10^4^/well) were seeded into 96-well plates and incubated for 24 h. Cells were treated with free C_60_ and Ber or C_60_-Ber nanocomplexes in 10 µM Ber-equivalent concentration for 0, 1, 3, 6, 12, and 24 h. Activity of caspase 3/7 was determined using the Promega Caspase-Glo^®^ 3/7 Activity assay kit (Madison, WI, USA) according to the manufacturer’s instruction. Briefly, plates were removed from the incubator and allowed to equilibrate to room temperature for 30 min. After treatment, an equal volume of Caspase-Glo 3/7 reagent containing a luminogenic peptide substrate was added followed by gentle mixing with a plate shaker at 300 rpm for 1 min. The plate was then incubated for 2 h at room temperature. The luminescence intensity of the products of caspase 3/7 reaction was measured with the Tecan Infinite M200 Pro microplate reader (Männedorf, Switzerland).

### 2.14. Statistics

All experiments were carried out with a minimum of four replicates. Data analysis was performed with the use of the GraphPad Prism 7 (GraphPad Software Inc., San Diego, CA, USA). Paired Student’s t-tests were performed. Differences values *p* < 0.01 were considered to be significant. Half-maximal inhibitory concentration (IC_50_) value was calculated with specialized software GraphPad Prism 7 (GraphPad Software Inc.). Individual concentration-effect curves were generated by fitting the logarithm of the compound concentration versus the corresponding normalized cell viability using nonlinear regression.

## 3. Results

### 3.1. Characterization of the C_60_-Ber Nanocomplex Aqueous Solution

**Figure 3:**
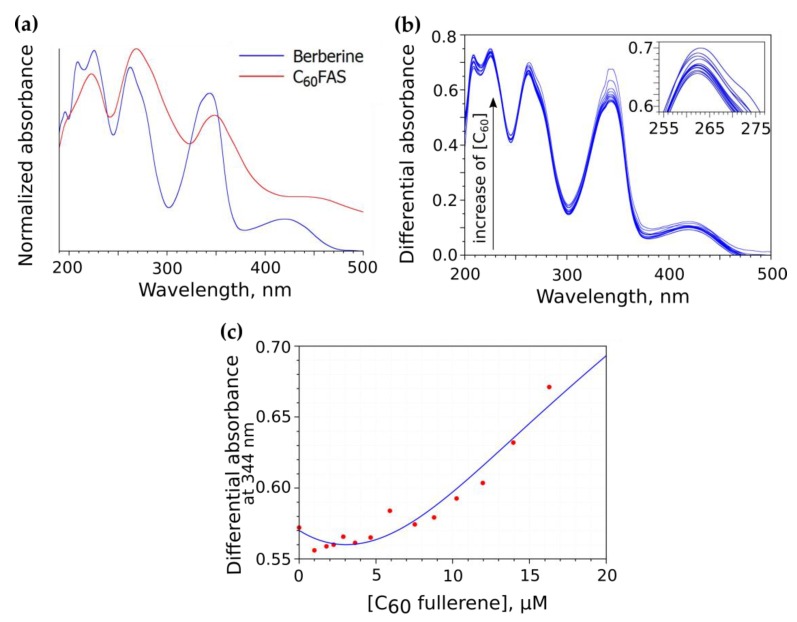
Absolute absorption spectra of Ber and C_60_ (**a**), differential absorption spectrum of C_60_-Ber nanocomplex solution (**b**), and the differential absorbance measured at absorption maximum of Ber molecule and fixed Ber concentration, *C_D_*_0_ = 0.02 mM, as a function of C_60_ concentration (**c**).

The initial test for possible interaction between C_60_ and Ber molecules was accomplished by means of UV–Vis spectroscopy. The Ber molecule exerts an absorption maximum at 344 nm that is overlapped with C_60_ absorption peaks, which does not allow for the unambiguous tracking of changes in the Ber spectrum upon the addition of C_60_ (Figure 3a). For that reason, we measured a differential absorption spectrum as a difference between the absolute spectrum of the C_60_-Ber nanocomplex and the absolute spectrum of pure C_60_FAS at the same C_60_ concentration. It can be seen that an increase in C_60_ concentration resulted in a non-monotonic change in the Ber spectrum with a slight bathochromic shift of the absorption maximum (Figure 3b). Very similar spectral indices have been previously noted for the related aromatic ligands, such as Doxorubicin [57,59], proflavine, and methylene blue [57], confirming nanocomplex formation with C_60_ in water. Moreover, the observed changes in the Ber spectrum appeared to be similar to that reported recently for the C_70_ and Ber complex [70]. These results evidence the formation of noncovalent nanocomplexes between C_60_ and Ber molecules in an aqueous solution.

The Ber absorption change (measured at 344 nm) as a function of C_60_ concentration is shown in Figure 3c. An increase in C_60_ concentration was followed by a systematic increase in the optical density. This repeated the trendline of the titration curve measured at elevated C_60_ concentrations for other ligand molecules [57] and was previously explained as a consequence of ligand adsorption into large C_60_ clusters with a further induction of C_60_ aggregation and a corresponding increase of light scattering. In the case of Ber, a similar effect could in principle be expected due to the Ber positive charge, which could attenuate the electrostatic repulsion between C_60_ molecules inside C_60_ clusters by analogy with a well-known effect of induced C_60_ aggregation in the presence of salts [71].

A preliminary AFM control study included an investigation of layers formed with free Ber and C_60_ to ensure the following proper interpretation of the data obtained with the C_60_-Ber layers.

**Figure 4:**
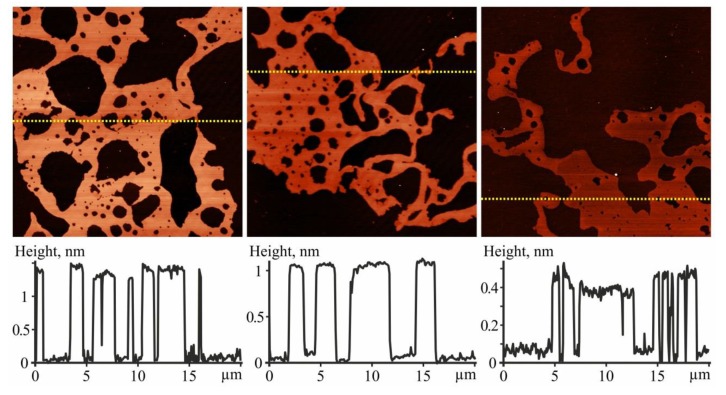
AFM images of the Ber (concentration 208 µM) layer (**top**) and their Z-profiles along the lines marked on images (**bottom**); image size is 20 × 20 µm^2^.

Ber layers contained molecules grouped in islands on the surface of the substrates. With the increased concentration of the deposited substance, a growth of the continuous submonolayer film was detected (Figure 4). AFM estimated the thickness of the Ber islands and submonolayers to be within a range of 0.35–2.2 nm. The smallest thickness of the submonolayer was found to be in good agreement with the minimum size of the Ber molecule: −1.47 × 0.66 × 0.32 nm [72]. Within one raster scanning, the thickness of the Ber submonolayer differed by no more than ~0.35 nm, which could be explained by its dependence on the number of molecular layers, the orientation of molecules in the submonolayer, the surface concentration of Ber molecules at a particular site, and the condition of their deposition. The observed formation of close-packed Ber islands and of submonolayers on the surface of the substrate indicates the attraction forces in the interaction between Ber molecules.

**Figure 5:**
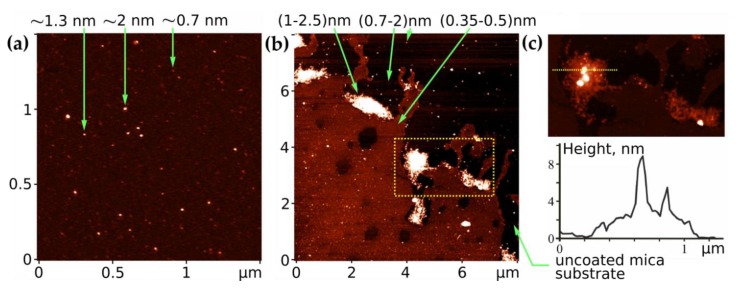
AFM images: the C_60_ (concentration: 208 µM) layer (**a**); the C_60_-Ber (208:208 µM) layer (**b**). Numbers with arrows show the height of nano-objects. (**c**) A 3.5 × 2 µm^2^ fragment (highlighted in image (**b**)) with reduced contrast (top) and its Z-profile along the marked line (bottom).

The investigation of C_60_ films deposited from an aqueous solution revealed a high degree of molecule dispersion in the solution. Therefore, the majority of C_60_ molecules were located chaotically and separately along the surface (see the dotted objects with a height of ~0.7 nm in Figure 5a), or in the form of bulk clusters consisting of several molecules (objects with a height of 1.3–2 nm in Figure 5a). The arrangement of C_60_ molecules formed because of the electrostatic repulsion between them. The observed formation of bulk nanoclusters in an aqueous solution is in excellent agreement with the results of our previous experimental and theoretical studies [39,40].

The characterization of the C_60_–Ber films was challenging due to the close proximity of sizes of the single C_60_ and Ber molecules. Therefore, the diameter of C_60_ (~0.7 nm) is equal to the size of the Ber molecule along one axis and double the size along the other axis. However, the different nature of the intermolecular interaction of free C_60_ and Ber molecules determines the different types of adsorption of these substances, namely the island-like growth of the Ber film and the isolated arrangement of C_60_ (nanoclusters), which can be used to identify C_60_ and Ber molecules in AFM images. As seen in Figure 5b, the continuous submonolayer was presented in the layer of the nanocomplex system, typical for Ber films (its thickness is 0.35–0.5 nm). The single objects were detected as well, which we identified as C_60_ molecules or their nanoclusters (0.7–2 nm). In the layers of the nanocomplex system, we observed conglomerates with a height of a few nanometers and a length of up to 1 μm (Figure 5b,c), absent in the layers of free C_60_ and Ber.

Therefore, it can be assumed that these conglomerates are a mixture of C_60_ and Ber molecules. This is indicated by the internal structure of the conglomerate, consisting of nanoaggregates of various types: A separate height of 1–2.5 nm, against the background of which there are one or several granules with a height of up to 10 nm (Figure 5c). The origin of conglomerates can be explained by the fact that the interaction negatively charged C_60_ nanoclusters [73], and Ber + cations in an aqueous dispersion are accompanied by their coagulation. Upon deposition on the surface and evaporation of water, a segregation of the nanocomplex occurs at the initial components with the formation of nanoaggregates of various types with van der Waals intermolecular interactions. It should be noted that our previous studies of the structural self-organization of C_60_ nanocomplexes with anticancer drugs Doxorubicin and Cisplatin in the physiological buffer showed that saline ions interfered with the C_60_ coagulation [58,59]. Therefore, to reduce the degree of aggregation of the C_60_-Ber nanocomplex, the development of this nanosystem in a physiological buffer could be promising.

At the same time, DLS measurements show the presence of large particles in the studied samples (Table 2). Additional aggregation of C_60_ after the addition of Ber molecules is clearly seen, which demonstrates the shift of the mean hydrodynamic diameter of light scattering particles to higher values, i.e., from 82 up to 152 nm. With an increase in Ber molecule content, the size of the particles in the studied C_60_–Ber dispersions was increased from 110 to 152 nm. A similar increase in aggregate size upon the addition of ligand molecules and complex formation has been previously reported for Doxorubicin [59], ICR-191 [53], and Landomycin A [61].

The PDI value, as an indicator of particles’ aggregation in an aqueous medium, was found to be in the range of 0.42–0.48 for the C_60_–Ber complexes (Table 2), indicating a high polydispersity of the studied aqueous dispersions.

The value of the zeta potential was determined to estimate the stability of nanocomplex dispersions. The zeta potential value for the studied C_60_-Ber nanocomplexes at room temperature changed from −19.51 to −21.26 mV (Table 2). This may be explained by Ber cation complexation with a negatively charged C_60_ and its nanoclusters in the aqueous dispersion (−23.9 mV).

**Figure 6:**
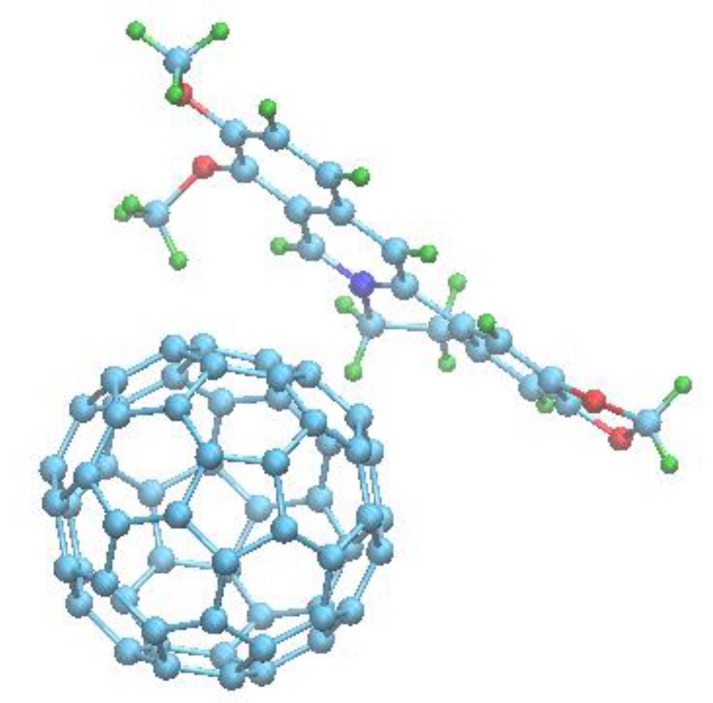
The energy-minimized structure of 1:1 C_60_-Ber nanocomplex.

The results presented above confirmed the complexation between the Ber molecule and C_60_. We then estimated the properties of the 1:1 nanocomplex by analyzing its energy-minimized structure (Figure 6). The structure reflects a face-to-face orientation of the Ber and C_60_ molecules’ aromatic surfaces, with a minimal distance between them of 0.328 nm. This well agrees with the structure of the 1:1 C_70_–Ber nanocomplex reported by Kyzyma et al. [70].

The structural analysis suggested that π-stacking should play a major role in nanocomplex stabilization in an aqueous solution [74]. The computation of the component’s total binding energy demonstrated that the net van der Waals (Δ*G_vdW_* ≈ −4.1 kcal/mol) and hydrophobic (Δ*G_hyd_* ≈ −9.6 kcal/mol) contributions are the leading factors favoring nanocomplex formation, whereas the contribution of electrostatic energy is small (Δ*G_el_* ≈ 1.3 kcal/mol). A similar pattern of the component’s binding energy was noted for various ligand molecule binding with C_60_ [74] and may therefore be viewed as a “thermodynamic signature” of C_60_ complexation with small aromatic molecules.

### 3.2. Computation of the Equilibrium Constant of Ber Binding with C_60_ Nanoparticles

In order to estimate the affinity of the Ber molecule to C_60_ nanoparticles in an aqueous dispersion, we used a standard approach in which the experimental titration curve (Figure 3c) was fitted using the complexation model, yielding the equilibrium complexation constant as an output search parameter [75]. Previously standard hetero-association models were shown to be not directly applicable for quantifying C_60_-ligand complexation by means of UV–Vis spectroscopy because ligand-induced C_60_ aggregation leads to pronounced light scattering that strongly influences the titration curve (see the discussion above). A general up-scaled model of C_60_-ligand complexation based on UV–Vis titration data has been recently suggested [60], and it takes into account the effect of ligand-induced C_60_ aggregation and the two major processes of ligand binding with C_60_ nanoparticles, i.e. ligand complexation with low-dimension C_60_ clusters with equilibrium constant *K_h_*_1_ (Process 1) and ligand adsorption into large C_60_ clusters with equilibrium constant *K_h_*_2_ (Process 2). In this model, the absorbance *A*, as a function of C_60_ concentration, *C*_0_, is given as

(2) $$A=\varepsilon_{m}C_{D1}^{\prime }+\varepsilon_{h1}BK_{h1}C_{R1}^{\prime }C_{D1}^{\prime }+\varepsilon_{h2}(C_{D0}-C_{D1}^{\prime }-BK_{h1}C_{R1}^{\prime }C_{D1}^{\prime })$$

where ε*_m_*, ε*_h_*_1_, and ε*_h_*_2_ are extinction coefficients of the ligand in a monomer state, in a complex with a low dimension and large C_60_ clusters, respectively (ε*_m_* equals 22,500 M^−1^·cm^−1^ for the Ber molecule [76], $C_{D0}$ is the ligand concentration, and $C_{R1}^{\prime }$ and $C_{D1}^{\prime }$ (as well as $C_{M0}^{\prime }$ (see [60] for details)) can be determined from the solution of the system of equations.

(3) $$\begin{array}{l} \left\{ \begin{array}{l} C_{0}=BC_{R1}^{\prime }+BK_{h1}C_{R1}^{\prime }C_{D1}^{\prime }+C_{M0}^{\prime }\frac{1-(1-B)BK_{F}C_{R1}^{\prime }}{1-BK_{F}C_{R1}^{\prime }}\\ C_{D0}=C_{D1}^{\prime }+BK_{h1}C_{R1}^{\prime }C_{D1}^{\prime }+C_{M0}^{\prime }\frac{HK_{h2}C_{D1}^{\prime }}{1-K_{h2}C_{D1}^{\prime }} \end{array}\right. \end{array}$$

where *K_F_* = 56,000 M^−1^ and *B* = 0.914.

Unknown parameters in this model are [ε*_h_*_1_, ε*_h_*_2_, *K_h_*_1_, *K_h_*_2_, *H*], which are determined by a standard numerical procedure of the minimization of discrepancy between the model (Equation (2)) and experimental (Figure 3c) titration curves (i.e., the curve fitting procedure).

In the present work, the above-described approach was used in a numerical analysis of Ber complexation with C_60_ in an aqueous dispersion. Table 3 contains the magnitudes of the examined parameters, obtained with the goodness of fit *R*^2^ = 0.94, evidencing the appropriateness of the model used.

Analysis of the complexation parameters enables to estimate the specificity of the binding process. Process 1 is characterized by the high *K_h_*_1_ magnitude and low molar absorption magnitude ε*_h_*_1_, as compared with the monomer molar absorption (i.e., ε*_m_* > ε*_h_*_1_). This indicates stacking complexation as a main form of Process 1, correspondent to the calculated structure presented in Figure 6. Process 2 is characterized by a low *K_h_*_2_ magnitude and high molar absorption magnitude ε*_h_*_2_, as compared with ε*_m_*. This evidences the non-specific adsorption of Ber molecules into large C_60_ clusters as a main form of Process 2. The resultant parameters are qualitatively similar to that obtained before for the binding of C_60_ in water with Doxorubicin and proflavine [60], both of which resemble Ber in terms of structure and charge state. Thus, the dominating event for binding Ber to C_60_ in water appears to be Process 1 (π-stacking).

### 3.3. Ber Release from C_60_-Ber Nanocomplexes

**Figure 7:**
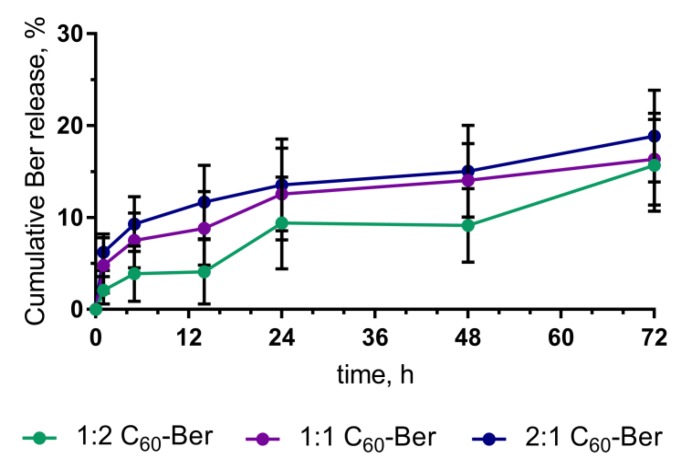
Berberine release from C_60_-Ber nanocomplexes during 72 h of incubation in RPMI medium.

Drug release is an important property of a therapeutic system, constituting a prerequisite to its biological application. To study the Ber release kinetics, C_60_-Ber nanocomplexes were incubated in the complex cell culture medium over 72 h. At 0, 1, 5, 14, 24, 48, and 72 h, the content of unbound free Ber was assessed with HPLC-ESI-MS/MS. Data obtained from in vitro drug release were plotted as cumulative amount of drug release versus time (Figure 7).

Drug release from from 1:2, 1:1, and 2:1 C_60_-Ber nanocomplexes under common cell culture conditions, was calculated to reach maximum of 15.68 ± 4.86%, 16.35 ± 5.07% and 18.87 ± 5.29% correspondingly of the initial concentration at 72 h of incubation (Figure 7). Thus, the content of 1:2, 1:1, and 2:1 C_60_-Ber nanocomplexes after incubation in RPMI medium for 72 h remained on the level of ≥85%.

### 3.4. Intracellular Accumulation of C_60_-Ber Nanocomplexes

**Figure 8:**
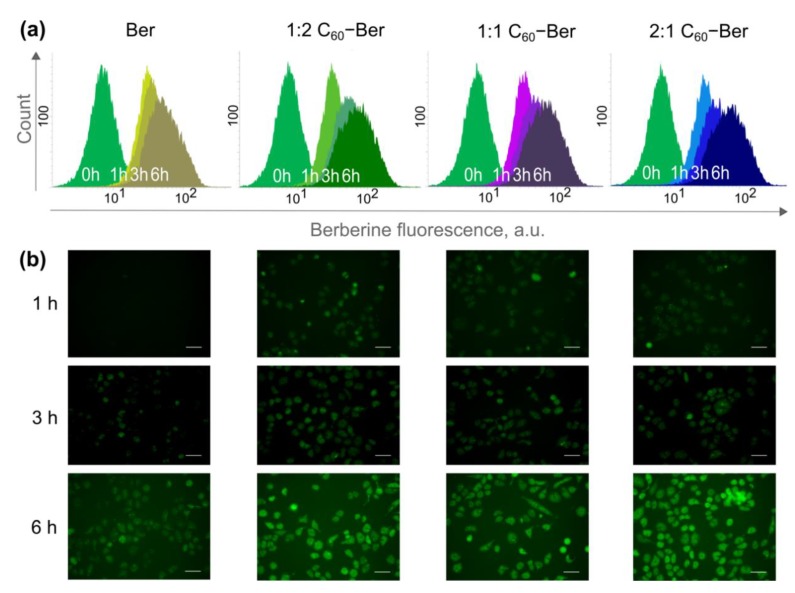
Intracellular accumulation of the free 10 µM Berberine and C_60_–Ber nanocomplexes in a Ber-equivalent concentration: flow cytometry (**a**) and fluorescent microscopy (**b**) of CCRF-CEM cells incubated with Ber and C_60_-Ber nanocomplexes at the molar ratios 1:2, 1:1, and 2:1; scale bar: 20 µm.

Strong absorption (Figure 3a) and fluorescence [20] of the Ber molecule in the visible spectral region enables the tracking of its complexes with the non-invasive, direct fluorescent-based techniques. CCRF-CEM cells were incubated in the presence of 10 µM Ber or C_60_-Ber nanocomplexes for 0, 1, 3, and 6 h and were examined with both fluorescent microscopy and flow cytometry to visualize and quantify the intracellular Ber uptake (Figure 8). Autofluorescence of the untreated cells was used as a negative control. The mean fluorescence intensity of each sample, calculated from logarithmic FACS histograms by the respective value of Ber green fluorescent signal (λ_ex_ = 488 nm, λ_em_ = 530/40 nm), is presented in Table 4.

Fluorescent microscopy demonstrated a time-dependent accumulation of 10 µM Ber in CCRF-CEM cells (Figure 8b). According to the literature data, Ber was localized in mitochondria [6] and effectively bound DNA, suggesting its high nuclear affinity [17,21].

Once Ber was complexated with C_60_, the observed fluorescence intensities were dramatically enhanced. Microscopy images demonstrated that C_60_-Ber nanocomplexes were internalized faster and more efficiently in comparison with the free Ber (Figure 8b). The mean fluorescent intensity of the CCRF-CEM cells (Figure 8a), treated with the 1:2 C_60_-Ber nanocomplex at 10 µM Ber-equivalent concentration, was found to be increased by 31% at 6 h. In cells treated with C_60_-Ber nanocomplexes at 1:1 and 2:1 molar ratios, the fluorescent signal reached the level of 130 and 140% from the control at 3 and 6 h, respectively (Table 4). The data obtained showed that Ber complexation with C_60_ strongly promoted its uptake by the leukemic cells.

### 3.5. Cell Viability

**Figure 9:**
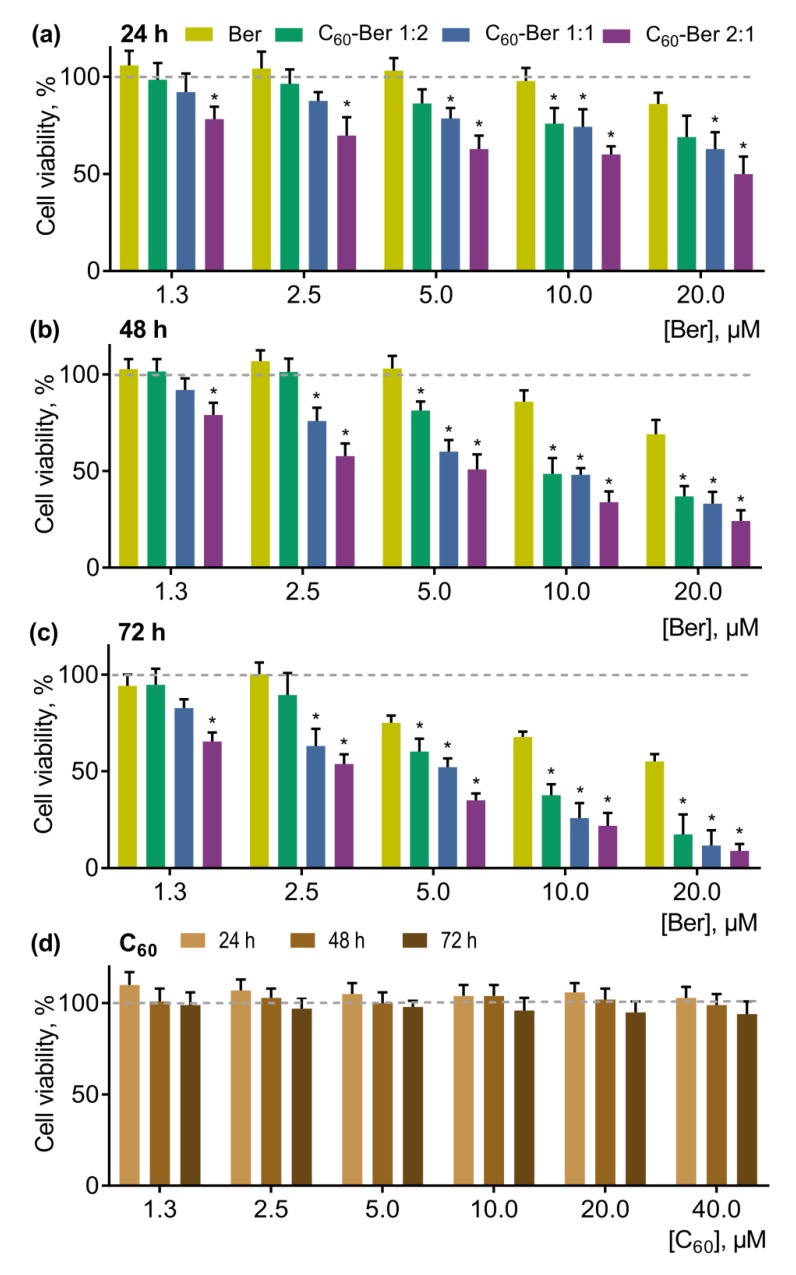
Viability of CCRF-CEM cells, treated with a free Ber or C_60_-Ber nanocomplexes in a Ber-equivalent concentrations for 24 (**a**), 48 (**b**), and 72 h (**c**) (* *p* ≤ 0.01 in comparison with the free Ber) as well as viability of CCRF-CEM cells, treated with a free C_60_ in a nanocomplex-equivalent concentrations for 24, 48, and 72 h (**d**).

To evaluate the effect of Ber on cancer cell proliferation, CCRF-CEM cells were treated with a free Ber in increasing concentrations and C_60_-Ber complexes in Ber-equivalent concentrations for 24, 48, and 72 h. Cell viability was estimated with an MTT assay (Figure 9).

Free Ber exhibited dose- and time-dependent toxicity towards CCRF-CEM cells in a range of concentrations from 5 to 50 µM (data are not shown). Taking into account the initial aim of anticancer agent complexation with C_60_ to potentiate its toxicity and, therefore, decrease efficient dose, we have chosen a concentration range—from 1.3 to 20 µM—for further investigation of the effects of Ber’s complexation with C_60_. The pointed concentrations of Ber exhibited mild, if any, cytotoxicity (Figure 9a–c).

Increasing concentrations of Ber inhibited cell growth in a time- and dose-dependent manner (Figure 9a–c). The number of viable cells was gradually decreased under the action of Ber in the concentration range 1.3–20 µM. Thus, 10 µM Ber decreased CCRF-CEM cell viability to 71 ± 9% and 50 ± 6% from the control at 48 and 72 h, respectively.

All C_60_-Ber nanocomplexes exhibited stronger antiproliferative potential towards CCRF-CEM cells in comparison with the free Ber. It should be noted that C_60_ alone at concentrations equivalent to those used in nanocomplexes had no significant effect on cell viability (Figure 9d). With the increasing of C_60_ concentration in C_60_-Ber nanocomplexes, a higher toxic potential towards CCRF-CEM cells was observed, following the order 1:2 < 1:1 < 2:1 (the most toxic). Thus, at 24, 48, and 72 h, the 10 µM 1:2 C_60_-Ber nanocomplex decreased cell viability to 76 ± 8%, 49 ± 8%, and 26 ± 7%; the 10 µM 1:1 C_60_-Ber nanocomplex to 74 ± 9%, 48 ± 3%, and 25 ± 7%; and 10 µM the 2:1 C_60_-Ber nanocomplex to 60 ± 4%, 34 ± 6%, and 22 ± 7% - all from the control, respectively (Figure 9a–c). The calculated IC_50_ values for the free Ber and C_60_-Ber nanocomplexes, listed in Table 5, evidenced the C_60_-dependent enhancement of Ber cytotoxicity. Thus, at 24 h, the IC_50_ value for Ber after complexation with C_60_ at the molar ratios 1:2, 1:1, and 2:1 was decreased by 1.3, 1.8, and 2.8 times, respectively; at 48 h, by 2.1, 2.9, and 4.6 times, respectively; at 72 h, by 3.2, 4.8, and 6.3 times, respectively.

### 3.6. Apoptosis Induction

**Figure 10:**
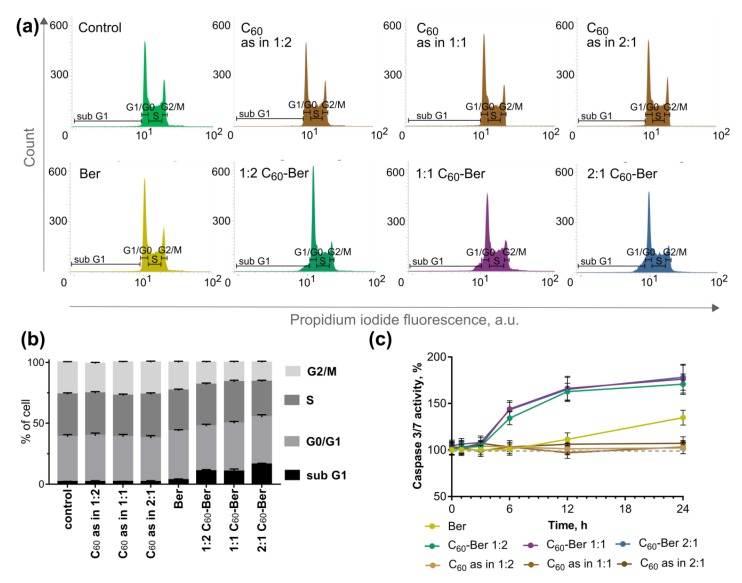
Apoptosis induction: cell cycle analysis in CCRF-CEM cells, incubated for 24 h after treatment with and without free C_60_, Ber or C_60_-Ber nanocomplexes at 10 µM Ber equivalent concentration presented with flow cytometry histograms (**a**) and a bar graph (**b**), which depicts the mean percentage of each cell cycle phase; caspase 3/7 activity in CCRF-CEM cells (**c**), incubated for 24 h after treatment with either free C_60_, Ber, or C_60_-Ber nanocomplexes at 10 µM Ber equivalent concentration (caspase 3/7 activity of untreated cells is set to 100%).

To determine whether C_60_-Ber nanocomplexes induced apoptosis, we monitored cell cycle distribution and caspase 3/7 activity in CCRF-CEM cells (Figure 10).

The analysis of flow cytometric cell cycle distribution indicated the accumulation of cells in the sub G1-phase upon complexation of Ber with C_60_. Thus, control cells were characterized with 1.94 ± 0.51% cells in the sub G1-phase. C_60_-treatment of control cells caused no alteration in the cell cycle distribution at three tested concentrations equivalent to C_60_-Ber nanocomplexes. CCRF-CEM cells treated with 10 µM Ber for 12 h showed a slight increase in sub G1-cells to 3.48 ± 0.89%. The treatment of CCRF-CEM cells with 1:2, 1:1, and 2:1 C_60_-Ber nanocomplexes was followed with an increase in cells in the sub G1-phase to 10.79 ± 1.21, 10.53 ± 2.01, and 16.28 ± 0.98%, respectively (Figure 10a,b).

Free C_60_ had no effect on the caspase 3/7 activity at the concentrations used in the nanocomplexes. In cells treated with the free Ber, a delayed caspase 3/7 activation by 35 ± 8% from the control at 24 h was observed. However, in cells treated with C_60_–Ber nanocomplexes, caspase 3/7 activation was detected starting from 6 h, which reached 170–178 ± 11–14% of the control at 24 h (Figure 10c).

The activation of caspase 3/7 cells indicated the induction of the apoptotic death of CCRF-CEM cells treated with C_60_-Ber nanocomplexes.

## 4. Discussion

The constantly increasing interest in novel nanotechnology platforms for biomedical applications stimulated the investigation and application of carbon nanomaterials including C_60_ as a representative of the fullerenes family. The supramolecular self-assembly based on π–π stacking interactions between unsaturated (poly)cyclic molecules is commonly used for the fast, easy, and cost-effective coupling of cargo molecules with carbon nanoparticles [77,78] and for improving drug stability and loading capacity [79].

In previous studies, we exploited the ability of the polyaromatic C_60_ surface to absorb different therapeutics and created the C_60_ drug-carrying nanocomplexes. In a pioneering attempt, Evstigneev et al. [57] showed a simple and fast method of noncovalent C_60_ complexation with Doxorubicin in water and later in a physiological solution [59]. The strategy was applied for C_60_ complexation with other chemotherapeutic drugs including Cisplatin and Landomycin A. Molecular modeling, spectroscopy, atomic-force microscopy, mass spectrometry, dynamic light, and small-angle X-ray/neutron scattering evidenced nanocomplex formation [54,56,59,60,80]. The proposed nanosystems were shown to have a higher toxicity compared with the free drugs in vitro and in vivo [54].

In the current study, nanocomplexes of C_60_ and the herbal alkaloid Ber at the 1:2, 1:1, and 2:1 molar ratios were prepared, characterized, and tested on human leukemic cells in vitro.

UV–Vis spectroscopy revealed that an increase in C_60_ concentration was followed by a non-monotonic change in the Ber spectrum with a slight bathochromic shift in its absorption maximum, which was induced by the ligand adsorption into large C_60_ clusters. C_60_-Ber nanocomplexes were prepared in the 1:2, 1:1, and 2:1 molar ratios. The increase in Ber concentrations in C_60_-Ber nanocomplexes was followed by the gradual increase in the particle size from 110 to 152 nm and in the zeta potential value from −21.26 to −19.51 mV, which was linked to the Ber-induced C_60_ aggregation and complexation of Ber cations with negatively charged C_60_. Finally, an AFM study indicated the internal structure of the 1:1 C_60_-Ber aqueous dispersion, consisting in particular of small nanoaggregates with a height of 1–2.5 nm.

The size of the proposed nanocomplexes could be classified as advantageous in that they are efficiently taken up by cancer cells, since the literature evaluation and discussion so far suggest an optimum size around 100–200 nm. This range is limited by the leaky tumor vessels, with the higher extravasation of macromolecules in a 10–500 nm size from one side [81,82,83,84], and the lymphatic system activation and the quick removal from a circulation of ≥200 nm nanoparticles from another side [85].

Finally, the computer simulation revealed that π-stacking was the dominating event in Ber and C_60_ binding in aqueous dispersions and allowed for the proposal of the energy-minimized structure of the 1:1 C_60_–Ber nanocomplex with a 1.05 nm minimum distance from C_60_ to the Ber nitrogen atom and 1.42 nm maximum distance from C_60_ to the Ber hydrogen atom. The obtained analytical data and the analysis of the Ber and C_60_ complexation parameters indicated the nanocomplex formation and their stability for in vitro studies.

With the use of the fluorescent microscopy and the flow cytometry, we confirmed the intracellular accumulation of the alkoloid in human leukemic CCRF-CEM cells treated with the free Ber or C_60_-Ber nanocomplexes. C_60_-Ber nanocomplexes appeared to be internalized by CCRF-CEM cells faster and more intensively then the free Ber. The intracellular Ber accumulation is determined by two independent processes—Ber entry into the cell and its efflux from the cell. Free Ber permeates the cells through the passive diffusion [86], while C_60_ enters the cell not only due to passive diffusion [87], but also by endocytosis/pinocytosis [88,89] and phagocytosis [90]. Therefore, C_60_ could function as a transporter of the small aromatic molecules [53], facilitating its intracellular uptake. From the other hand, Ber was reported to be a substrate of P-glycoprotein [91], responsible for the fast pumping of drugs from the cancer cell. However, C_60_ is not recognized by P-glycoprotein [92] and is even shown to bind P-glycoproteins [55], inhibiting its activity. Taken together, these data indicate that the enhanced cytotoxic effect of C_60_-Ber nanocomplexes can be linked to the increased alkoloid’s accumulation in leukemic cells.

Cell viability assay revealed a time- and concentration-dependent toxic effect of Ber towards CCRF-CEM cells. IC_50_ values were estimated to be 58 ± 5, 23 ± 2, and 19 ± 2 µM at 24, 48, and 72 h, respectively. The IC_50_ of Ber in 1:2, 1:1, and 2:1 C_60_–Ber nanocomplexes was decreased at 72 h by 3.2, 4.8, and 6.3 times, respectively, following the order 1:2 ˂ 1:1 ˂ 2:1 (the most toxic). The activation of caspase 3/7 and altered cell cycle distribution in CCRF-CEM cells indicated the apoptotic cell death induction under C_60_-Ber nanocomplexes action.

The enhanced toxic efficiency of Ber against leukemic cells upon its complexation with C_60_ as compared with the free drug is determined by C_60_’s ability to promote drug accumulation inside cancer cells and potentiate its toxic activity. Complexation with C_60_ allowed us to enhance Ber toxicity against leukemic cells more appreciably as compared with C_60_ complexation with traditional anticancer therapeutic Doxorubicin, which was followed by a less than 3.5-fold decrease of IC_50_ at the same treatment duration [56]. This can be linked to the higher concentration of the safe Ber in the C_60_-containing nanocomplex (µM of Ber against nM of Doxorubicin). The obtained results encourage the strategy of C_60_ usage for natural anticancer medicine delivery.

## 5. Conclusions

In the presented study, the fast and easy noncovalent complexation strategy of aromatic cargo with C_60_ fullerene was used to deliver the herbal alkaloid Ber into leukemic cells.

The UV–Vis spectroscopy, DLS, and AFM techniques may confirm the complexation of C_60_ with the Ber molecule in an aqueous dispersion, and computer simulation allowed for the proposal of the energy-stable structure of 1:1 C_60_-Ber nanocomplex with a size of up to ~1.4 nm.

Fluorescence-based techniques evidenced that C_60_–Ber nanocomplexes were more quickly and more intensely internalized by leukemic CCRF-CEM cells and exhibited a stronger antiproliferative potential as compared with free Ber. The IC_50_ value for Ber in C_60_-Ber nanocomplexes at 1:2, 1:1, and 2:1 molar ratios was found to be decreased by 3.2, 4.8, and 6.3 times, respectively, as compared with the IC_50_ value for the free Ber. The activation of caspase 3/7 and increase of the sub-G1 cell cycle phase in CCRF-CEM cells indicated the apoptotic cell death induction under treatment with C_60_-Ber nanocomplexes.

The results of this study suggest the formation of a noncovalent nanocomplex between herbal alkaloid Ber and C_60_ fullerene. The complexation of Ber with C_60_, as a nanocarrier, enhanced its uptake by leukemic cells with toxic effects. Our results provide a proof of concept of the strategy of using C_60_ for natural medicine nanodelivery.

## Figures and Tables

**Table 1:** HPLC-ESI-MS/MS conditions for analysis of Ber.

**Chromatographic Conditions**	
Column, its temperature	Agilent Eclipse XDB-C18, 40 °C
Mobile phase	Acetonitrile:0.1% formic acid in H_2_O
Flow rate	0.7 mL/min
Run time	5 min
Injection volume	1 µL
**MS/MS Conditions**	
Desolvation line temperature	250 °C
Heat block temperature	400 °C
Target molecular ion	336.25 [M] + *m/z*
Product ions	321.20, 320.20, 292.25 *m/z*
Time window	0–5 min
Dwell time	0.2 s
Interface voltage	4.5 kV
Nebulizing gas flow	3 L/min
Drying gas flow	15 L/min

**Table 2:** The hydrodynamic diameter of particles, polydispersity index (PDI), and zeta potential values ^1^ for the studied samples at room temperature.

Sample	Size, nm	PDI	Zeta Potential, mV
1:2 C_60_-Ber	152 ± 2, (3…289) ^2^	0.44 ± 0.02	−19.5 ± 0.5
1:1 C_60_-Ber	114 ± 2, (3…207) ^2^	0.42 ± 0.02	−20.6 ± 0.5
2:1 C_60_-Ber	110 ± 2, (3…196) ^2^	0.48 ± 0.02	−21.3 ± 0.5
C_60_FAS	82 ± 2, (3…126) ^2^	0.21 ± 0.01	−23.9 ± 0.6

^1^ Results are presented as mean values ± SD; ^2^ The minimum and maximum particle sizes are given in parentheses.

**Table 3:** The calculated parameters of Ber complexation with C_60_ in an aqueous dispersion.

ε*_h_*_1_, M^−1^·cm^−1^	ε*_h_*_2_, M^−1^·cm^−1^	*K_h_*_1_, M^−1^	*K_h_*_2_, M^−1^	*H*
4250	46,100	28,300	4300	22

**Table 4:** Mean fluorescence intensity (FI) of the intracellular accumulated Ber measured with flow cytometry.

FI, a.u.	1 h	3 h	6 h
Ber	39 ± 3	45 ± 3	57 ± 5
1:2 C_60_-Ber	38 ± 2	49 ± 4	80 ± 7 *
1:1 C_60_-Ber	42 ± 4	59 ± 5 *	79 ± 6 *
2:1 C_60_-Ber	38 ± 3	57 ± 6 *	81 ± 6 *

* *p* ≤ 0.01 in comparison with the free Ber.

**Table 5:** Half-maximal inhibitory concentration (IC_50_) of the free Ber and C_60_-Ber nanocomplexes towards CCRF-CEM cells.

IC_50_, µM	24 h	48 h	72 h
Ber	58 ± 5	23 ± 2	19 ± 2
1:2 C_60_-Ber	44 ± 4 *	11.0 ± 1.2 *	6.0 ± 0.4 *
1:1 C_60_-Ber	33 ± 3 *	8.0 ± 0.7 *	4.0 ± 0.3 *
2:1 C_60_-Ber	21 ± 2 *	5.0 ± 0.6 *	3.0 ± 0.2 *

* *p* ≤ 0.01 in comparison with the free Ber.
